# Foreign language anxiety research in *System* between 2004 and 2023: looking back and looking forward

**DOI:** 10.3389/fpsyg.2024.1373290

**Published:** 2024-04-22

**Authors:** Qiangfu Yu

**Affiliations:** Faculty of Humanities and Foreign Languages, Xi’an University of Technology, Xi’an, Shaanxi, China

**Keywords:** foreign language anxiety, foreign language learning, English as a foreign language, foreign language learners, literature review

## Abstract

With the deepening of the research on emotional factors, foreign language anxiety (FLA) has become the focus of researchers in the field of foreign language learning (FLL) and teaching. This paper aims to provide an overview of the historical trajectory of FLA research that has been published in *System* between 2004 and 2023. While examining the retrieved 49 studies, focus has been laid on the methodologies including research instruments, methods, participants, major themes and key findings of FLA research. Although almost all of the studies employed quantitative and mix-methods methodologies, questionnaires and semi-structured interviews were the most preferred research methods. FL learners from 21 countries/regions were represented, but a significant number of the studies came from China, Japan and Iran. And an overwhelming majority of the studies focused on FLA among the learners learning English as a foreign language (EFL). The review concluded with some research lacunae and possible directions for future research on FLA.

## Introduction

FLA, prevalent among foreign language (FL) learners (Dewaele and Macintyre, 2014; Li, 2020), is a very special and complex psychological phenomenon during the process of FLL (Gardner, 1985; Macintyre and Gardner, 1994). FLA is regarded as the biggest emotional obstacle during the process of FLL (Arnold and Brown, 1999), which may undermine students’ confidence and motivation in FLL (Macintyre, 2017). Horwitz (2010) considered FLA as one of the strongest predictors of success or failure in FLL. Previously, anxiety in FFL, as an auxiliary variable in FLL research, had only drawn scarcity of attention from researchers (Chastain, 1975; Dewaele and Li, 2021). It was not until 1986 that Horwitz et al. (1986), for the first time, proposed the concept of FLA, reckoning that FLA is a unique synthesis of self-perception, belief, emotion and behavior associated with FLL. Meanwhile, Horwitz et al. (1986) developed the Foreign Language Classroom Anxiety Scale (FLCAS), which has become the most widely accepted FLA scale. Since then, researchers have conducted a plethora of studies on the connotations (Macintyre and Gardner, 1994; Oxford, 1999), categorization (Horwitz et al., 1986; Ellis, 1994; Macintyre and Gardner, 1994), impacts (Steinberg and Horwitz, 1986; MacIntyre and Charos, 1996), sources (Young, 1991; Macintyre, 2017), and measurement tools (Macintyre and Gardner, 1994; Satio et al., 1999; Kim, 2000; Elkhafaifi, 2005; Woodrow, 2006; Cheng, 2017) of FLA.

*System*, one of the most influential and prestigious international journals devoted to FL teaching and learning, has stayed abreast of the development of FLA research. The articles having been published on FLA in *System* represent to a large extent the development trajectory of FLA research. Therefore, this review paper chooses *System* as the material to provide the historical trajectory of FLA research and suggest some under-researched topics and future directions of FLA research.

## Foreign language anxiety

FLA, a principal learner emotional factor in foreign language learning (FLL), has become one of the significant research focuses in FLL since the 1970s. Originating from psychology, anxiety is defined as “an unpleasant state of mind that is characterized by individual perceived feelings like nervous, fear, and worry, and is activated by the autonomic nervousness system” (Spielberger, 1972). FLA is a unique form of anxiety in the specific context of foreign language learning (Horwitz et al., 1986; MacIntyre, 1995). Horwitz et al. (1986) conceptualized FLA as “a distinct complex of self-perceptions, beliefs, feelings, and behaviors related to classroom language learning arising from the uniqueness of the language learning process.”

Horwitz et al. (1986) first studied FLA as an independent phenomenon. In order to resolve the deficiency and insufficiency of traditional research tools in respect of FLA, Horwitz et al. (1986) framed the Foreign Language Classroom Anxiety Scale (FLCAS), putting an end to the history of FLA study having no standardized measurement tools (Guo and Xu, 2014), foreboding that FLA research entered a period of relative maturity when researchers began to focus on the overall performance of FLA and its relationship with a variety of variables (Young, 1986, 1992; Aida, 1994), as well as the relationship between FLA and some basic language skills like listening, speaking, reading and writing (Gungle and Taylor, 1989; Vogely, 1998; Sellers, 2000).

Simply put, FLA is the feeling of tension, fear and nervousness in self-consciousness, emotions, beliefs, and behaviors (Aida, 1994) associated with a context which requires an individual to use a foreign language he or she is not proficient with (MacIntyre and Gardner, 1991).

## Research design

In order to present a systematic analysis of FLA research published in *System*, a narrative approach of systematic review was adopted. Systematic review involves “a clearly formulated question” and adopts “systematic and explicit methods to identify, select, and critically appraise relevant research, and to collect and analyze data from the studies that are included in the review” (Cochrane Collaboration, 2003). A narrative approach relies “primarily on the use of words and text to summarize and explain the findings,” and is considered helpful to systematically review topics that have been studied differently researchers (Popay et al., 2006), highlight the strengths and limitations of studies being reviewed (Wong et al., 2013).

The review aims to provide a systematic analysis of FLA research during the past two decades between 2004 and 2023 by answering the following questions:

Question 1: What is the overall trend in FLA research published in *System* during the past two decades?

Question 2: What are the major themes and the key findings of FLA research?

Question 3: What are the existent gaps in the current research and the potential directions for future research?

### Data collection

**Figure 1:**
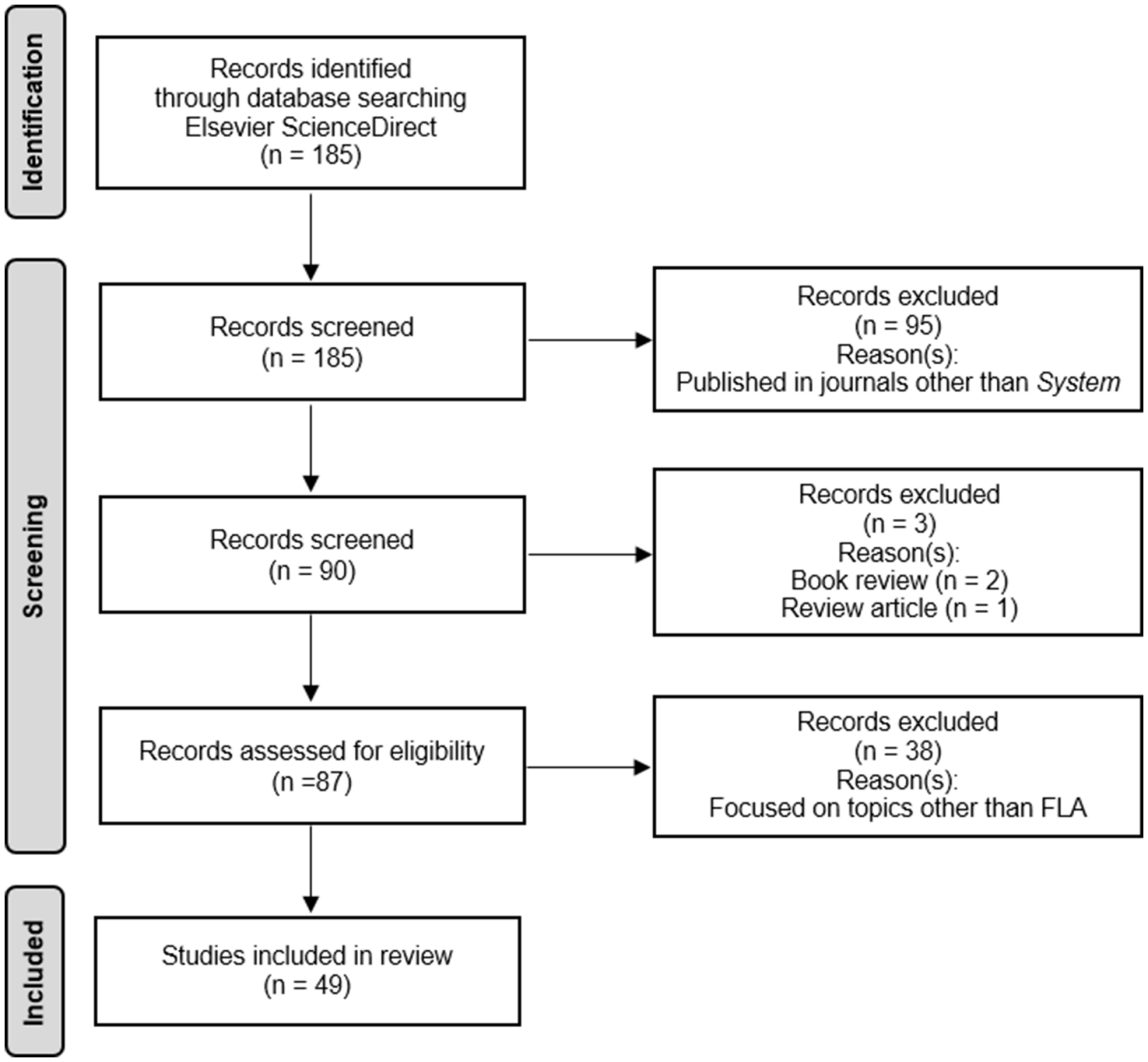
PRISMA flow chart.

Following the PRISMA guidelines (Moher et al., 2009), an extensive literature search was conducted to ensure a comprehensive analysis of the current FLA research published in *System*. The data selection criteria and collection process are summarized in Figure 1.

Firstly, relevant studies published until and including December 15, 2023 were searched in the database of Elsevier ScienceDirect. The author conducted advanced searches in the database with the following searching parameters: *In this journal or book title* = (*System*) AND *Title, abstract, or author-specific keywords* = (anxiety). Overall, the database returned 185 publications, among which 95 were published in journals other than *System* and therefore were deleted. Then, 2 book reviews and 1 review article were deleted. The remaining 87 publications were evaluated for the eligibility by reading and analyzing the titles, abstracts and full texts, and 38 publications were excluded based on the following criterion that the studies focused on topics other than FLA.

### Data analysis

This review first conducted a bibliometric analysis of the retrieved records. A coding analysis was then performed through iterative reading with the highlights on the following categories that guided the data analysis: year of publication, characteristics of samples, research methodologies, and key findings.

## Results

### Descriptive characteristics of studies

#### Publishing years

**Figure 2:**
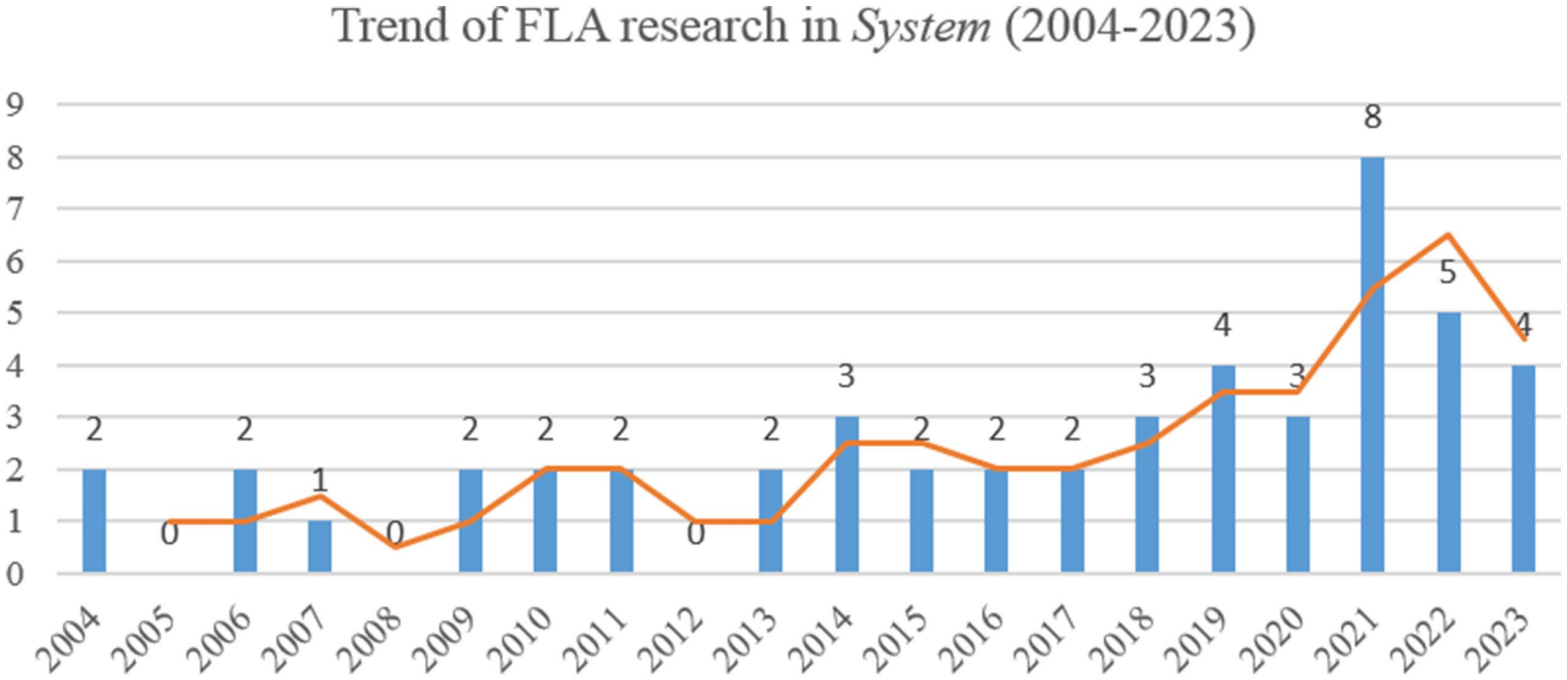
Trend of FLA research in *System* (2004–2023).

There is a dynamic upward trend in the number of studies on FLA over the past two decades (see Figure 2). 2021 witnessed a surge in the number of publications, reaching an all-time peak of 8 papers. There is a gradual downward trend in the following 2 years, but compared with the average of about 2 papers per year, there is still an increase in the number of papers in 2022 and 2023.

#### Countries/regions of research

There was diversity of countries/ regions where the studies took place, with 21 countries/regions represented. Twenty-one papers (42.86%) came from China, followed by 6 papers (12.24%) from Japan and 4 papers (8.16%) from Iran. Three papers were conducted in Korea and USA each, followed by 2 papers from Saudi Arabia, and 1 paper from Canada, Australia, Indonesia, Austria, Germany, Switzerland, Italy, Slovakia, Macau, Chile, Thailand, Turkey, and UK each.

#### Research participants

The overwhelming majority of the studies (*n* = 43, 87.76%) focused on FLA among university students, with 3 papers focused on primary school students and 5 on secondary school students. It is noteworthy to point out that there were 3 studies which focused on PhD students, adult students, and vocational high school students, respectively.

#### Foreign languages studied

Since the status of English as a universal language is beyond doubt, almost all the studies examined FLA in the context of EFL learning. Among the 49 papers, there were only 4 papers focusing on FLA in the context of FLL other than EFL learning. These foreign languages included Korean, Arabic, German and Chinese. There was 1 paper comparing the possible FLA differences between the contexts of German as L1 learning and English as LX learning.

#### Research methodologies

Of the retrieved records, 38 were cross-sectional studies and 11 were longitudinal studies. The average length of time for the longitudinal studies was approximately 11.36 weeks, ranging from the shortest length of 1 week to the longest length of 18 weeks. The studies adopting quantitative methods (*n* = 24, 48.98%) and the studies using mix-methods (*n* = 24, 48.98%) markedly dwarfed the only one study using qualitative methods (2.04%).

Questionnaires were the most common research method in both quantitative studies and mix-methods studies. The FLCAS was the most frequently-used scale (*n* = 22, 48.83%), which a significant number of studies used directly (*n* = 7, 14.58%), adapted (*n* = 4, 8.33%), modified (*n* = 6, 12.50%) or translated (*n* = 5, 10.42%). Besides, a number of studies used questionnaires that adopted, modified or translated other scales such as the FLRAS (Satio et al., 1999), the FLLAS (Elkhafaifi, 2005), and the S-FLCAS (Dewaele and Macintyre, 2014) among many others. Notwithstanding, some researchers devised targeted questionnaires (Hurd, 2007; Woodrow, 2011; Lee, 2016; Li, 2018; Wang H. et al., 2021; Alrabai, 2022).

With regard to qualitative methodology, the research methods frequently used to measure FLA embraced interviews (*n* = 15, 62.50%), classroom observations (*n* = 5, 20.83%), students’ reflective journals (*n* = 5, 20.83%), open-ended questions (*n* = 3, 12.50%). Of note, Hurd (2007) employed audio-recorded think-aloud protocols combined with questionnaires and one-to-one semi-structured telephone interviews to explore FLA in a distance learning environment. Dryden et al. (2021) used linguistic ethnography to investigate how four migrant EFL learners in Australia experienced FLA.

### Research themes and key findings

#### Level of FLA

Twelve papers (24.49%) were found to investigate FLA level of FL learners, however, no consensus has been reached on the level of FLA among FL learners, possibly due to the fact that the participants of the retrieved studies were different. For example, Jiang and Dewaele (2020) found 1,031 university freshmen in China experienced a moderate level of FLA. Zuniga and Simard (2022) and Lee et al. (2023) had similar findings. However, Jiang and Dewaele (2019) found a higher level of FLA among 564 EFL university freshmen in China than the counterpart participants in the study of Dewaele and MacIntyre (2014). Similarly, Bekleyen (2009) found the language teacher candidates in Turkey experienced a high level of FL listening anxiety.

Dynamicity of FLA drew attention from some scholars. Koga (2010) investigated the dynamicity of FLA among 88 first-year university students in Japan and found FLA decreased significantly at the end of the 15-week English courses. Veenstra and Weaver (2022) investigated 341 students from two private universities in Japan and a continuum of FL speaking anxiety showed that the participants’ overall level of FL speaking anxiety decreased after finishing an English presentation course lasting 15 weeks.

Some studies explored some potential differences of FLA among different participants or among the same participants in different contexts. For example, Chen et al. (2022) found Chinese undergraduates had a higher level of EFL reading anxiety than Spanish undergraduates. Resnik and Dewaele (2020) found the participants experienced a higher level of FLA in English (LX) classes than in German (L1) classes.

#### Sources of FLA

Nine studies (18.37%) explored sources or causes of FLA. Bekleyen (2009) revealed some major sources of FL listening anxiety, including low priority of listening in previous FLL, and failure to recognize the spoken form of word, phrase or sentence. Jiang and Dewaele (2019) uncovered a number of factors contributing to FL class anxiety, including exams and quizzes, speaking in front of the class without preparation, challenging classroom activities, and teacher questioning. Bashori et al. (2021) identified insufficient vocabulary knowledge as one of the factors provoking FL speaking anxiety. Besides, speaking strategies, willingness to communicate, speaking self-efficacy and speaking proficiency were found to have positive direct effects on FLA (Sun and Teng, 2021). Of note, Zare et al. (2022) focused on FLA outside the traditional face-to-face classroom and found that autonomous learning was the source of the participants’ anxiety during the data-driven FFL.

#### Correlation of FLA with other variables

Some studies (*n* = 5, 10.20%) explored the correlation of FLA with demographic variables of the participants. Park and French (2013) found female students had significantly higher levels of FLA than male students. However, Jiang and Dewaele (2020) found gender and ethnic affiliation were not correlated with FLA while geographical background and experience in traveling abroad had a weak correlation with FLA. Similarly, Matsuda and Gobel (2004) found EFL learners with overseas experience experienced lower anxiety when speaking English and gender did not have a significant effect on FLA. However, Yim (2014) found gender had a significant effect of FLA. The discrepancies in the correlation with demographic variables may be attributed to the different samples or the possibility that male learners are not inclined to willingly admit anxiety than female learners (Williams, 1996; Pappamihiel, 2002).

A number of studies (*n* = 9, 18.37%) explored the correlation of FLA with academic performance/ achievement. For example, Pyun et al. (2014) found that oral achievement of the participants was negatively correlated with FLA. However, Tsang and Lee (2023) found FL speaking anxiety was not significantly related to speaking proficiency. Hamada and Takaki (2021) found FL reading anxiety had significantly direct effects on course achievement. Woodrow (2011) and Li et al. (2023) found FL writing anxiety was significantly negatively correlated with writing performance, but FLA did not have a significant prediction on writing achievement (Li et al., 2023). Besides, In’nami (2006) found that test anxiety did not affect FL listening test performance.

Many studies (*n* = 19, 38.78%) focused on the correlation of FLA with other student-specific variables, including learning motivation (Tsai and Liao, 2021), willingness to communicate (Lee and Hsieh, 2019; Wang H. et al., 2021), language proficiency (Jiang and Dewaele, 2020) and trait emotional intelligence (Resnik and Dewaele, 2020; Li et al., 2021) among many others. Several studies (*n* = 5, 10.20%) focused on the correlation of FLA with teacher-specific variables, such as teachers’ oral corrective feedback (Lee, 2016), perceived teacher emotional support (Jin and Dewaele, 2018), and teaching styles (Briesmaster and Briesmaster-Paredes, 2015).

#### Ways to relieve FLA

Ways to relieve FLA was also a topic of immense interest to researchers. Ten studies (20.41%) explored how to relieve or alleviate FLA. Jin et al. (2021) and Alrabai (2022) applied positive psychology intervention to reduce leaners’ FLA. Alrabai (2022) revealed that the integration of positive and negative emotions in FLL could result in alleviation of FLA among Saudi EFL learners. Jin et al. (2021) uncovered that reminiscing about language achievements significantly mitigated the levels of FLA among Chinese EFL learners. Similarly, Lee et al. (2023) found that constructing learners’ growth language mindset relieved their FLA.

Besides, Tsai and Liao (2021) found using machine translation systems had a positive effect on lowering FLA among EFL learners in Taiwan. Bashori et al. (2021) investigated the potential effects of Automatic Speech Recognition-based websites on EFL learners’ vocabulary, FLA and FLE. Other studies found that self-regulatory strategies (Guo et al., 2018), recasts (Li, 2018), and translanguaging (Dryden et al., 2021) had a significant effect on mitigating the levels of FLA among EFL learners. Of note, Kralova et al. (2017) employed psycho-social training as a strategy to alleviate FLA among 68 Slovak EFL learners.

## Discussion

During the past two decades between 2004 and 2023, *System* has been an ardent supporter of FLA research, committed to probing into and resolving FLA-related problems of foreign language teaching and learning. However, based on the review, some research lacunae are discerned concerning samples, methodologies and themes of FLA research, and some possible directions for future FLA research are also suggested.

### Research samples

Notwithstanding the FLA studies in *System* involved a variety of FL learners as the participants, there was a serious polarization phenomenon concerning the diversity of the research samples. An overwhelmingly large number of the studies focused on FLA among the FL learners in school and few studies focused on FLA among non-school FL learners. Moreover, a majority of the studies explored FLA among undergraduate students, especially the non-English-major university students, and there is a scarcity of studies investigating FLA among students in primary schools, secondary schools, vocational colleges as well as postgraduate students. In terms of geographical distribution of the research samples, most studies focused on FL learners from Asian countries including China, Japan and Iran among many others, and less attention was paid to FL learners from Europe, North America and South America. And no studies on FLA involving FL learners in Africa have been found. Meanwhile, most participants were from urban places, and only a couple of studies explored FLA among rural FL learners (Hamada and Takaki, 2021; Li et al., 2023). Last but not the least, with regard to the types of FL, a plethora of studies concentrated on English as a FL. Of the 49 retrieved studies, only 4 studies focused on FLA among the participants learning Korean, Arabic, German and Chinese as a FL, respectively.

Future research should diversify the research objects and focus increasing attention on the FLA research among primary school students, secondary school students, vocational college students and non-school FL adult learners, and moderate attention should be paid to the FLA research among preschool children and postgraduate students, so as to avoid the polarization of research samples. Besides, the dominance of English as a *lingua franca* has made English the FL taught in schools around the globe (Rose et al., 2020), facilitating FLA studies among EFL learners. However, recent years has witnessed frequent calls for conducting research on teaching and learning of foreign languages other than English (Zhang et al., 2019; Guo et al., 2021). Future studies can also focus on FLA among learners of foreign languages other than English as well as FL learners in countries and regions outside Asia.

### Research methodologies

Notwithstanding an increasing number of studies combined quantitative methods and qualitative methods in recent years, questionnaires were still the staple tool for quantitative data collection, and semi-structured interviews for qualitative data collection. A few mix-methods studies used classroom observation, student journals, field investigation and case studies for qualitative analysis. In addition, the FLCAS was the most popular scale for quantitative data collection and analysis, and only a few studies devised target questionnaires for their research. Moreover, cross-sectional studies far exceeded longitudinal studies, and the average length of time for longitudinal studies were relatively short, lasing about 10 weeks. Finally, there were only three comparative studies on FLA, probing into FLA differences among the participants (Resnik and Dewaele, 2020; Hamada and Takaki, 2021; Chen et al., 2022).

Future FLA research should adopt mix-methods studies with qualitative research not just being confined to semi-structured interviews, but embracing a variety of methods, such as classroom observation, video recording, student journals, field investigation, case study and particularly audio-recorded think-aloud protocols. And path analysis and structural equation modeling analysis should be increasingly employed to analyze the quantitative data. Meanwhile, some advanced techniques such as Event-related Potentials (ERP), Positron Emission Tomography (PET) and functional Magnetic Resonance Imaging (fMRI) can be used in future research to analyze FLA from the perspective of neural mechanism by measuring the electromagnetic, blood flow and neuronal activities of the human brain. In addition, it is necessary to devise localized FLA scale with ideal validity and reliability in accordance with the cultural background and educational environment of the country or region where the research objects are located. Moreover, the dynamic nature of FLA requires more longitudinal studies to explain how FLA changes dynamically and what impacts FLA exerts on FLL. Finally, future studies can pay more attention to the comparative study of FLA differences among different groups, which is more conducive to understanding the characteristics and distribution of FLA among different groups of FL learners, so as to put forward targeted strategies to mitigate FLA in FLL.

### Research themes

Research themes of the studies on FLA in *System* were of rich variety. However, no research has been found on translation anxiety and interpretation anxiety. Besides, there was a scarcity of research on the effectiveness of alleviating FLA. Studies on strategies to reduce FLA were mostly conducted from the perspective of teachers, and few studies revealed how to alleviate FLA from the perspective of learners. And most of the specific strategies to mitigate FLA were only at the theoretical level, lacking sufficient theoretical and empirical evidence, which were not applicable in practical FL teaching.

The following research themes deserve more attention in future research: translation anxiety and interpretation anxiety, types and effectiveness of strategies for alleviating FLA among different groups of FL learners, FLA among learners of heritage languages as well as non-heritage languages, and comparative studies on the effects of regional locations and mother languages on FLA. Moreover, future studies should not only focus on the theoretical research of FLA, but also carry out more empirical studies on strategies on how to alleviate FLA among different FL learners, such as learners from different regional locations, learners in monolingualism, bilingualism and multilingualism, and the effectiveness of FLA-alleviating strategies.

## Conclusion

By reviewing the 49 studies on FLA published in *System* between 2004 and 2023, this paper demonstrates that the journal’s commitment to FLA research embraces a wide range of research themes being explored with different research methods. Based on the findings of the review, some research lacunae regarding samples, methodologies and themes of FLA research are discussed, and some possible directions for future FLA research are also suggested.

## Data availability statement

The original contributions presented in the study are included in the article/supplementary material, further inquiries can be directed to the corresponding author.

## Author contributions

QY: Writing – review & editing, Writing – original draft.
